# Communicating environmental exposure results and health information in a community-based participatory research study

**DOI:** 10.1186/s12889-018-5721-1

**Published:** 2018-06-25

**Authors:** Luz Claudio, Jalisa Gilmore, Mohana Roy, Barbara Brenner

**Affiliations:** 1 Department of Environmental Medicine and Public Health, Division of International Health, One Gustave L. Levy Place, #1057, New York, NY 10029-6574 USA; 2 Albert Einstein College of Medicine, Bronx, USA; 3 Beth Israel Deaconess Medical Center, Harvard Medical School, Boston, Massachusetts USA

**Keywords:** Report-back, Children’s environmental health, Study participants, Health disparities, Community-based participatory research, Exposure assessment, Environmental health literacy, Results communication, Risk communication

## Abstract

**Background:**

Communicating results to participants is a fundamental component of community-based participatory research (CBPR). However, in environmental exposure studies this is not always practiced, partly due to ethical concerns of communicating results that have unknown clinical significance.

**Methods:**

*Growing Up Healthy* was a community-based participatory research study that sought to understand the relationship between environmental exposures to phthalates and early puberty in young girls. After in-depth consultation with a Community Advisory Board, study investigators provided group summary results of phthalate exposures and related health information to the parents of study participants. Parents’ comprehension and knowledge of the health information provided was then assessed through questionnaires.

**Results:**

After receiving the information from the research team, responders were able to correctly answer comprehension questions about phthalate exposures in their community, were able to identify ways to reduce exposure to phthalates, and indicated plans to do so. Questionnaires revealed that parents wanted more information on phthalates, and that children’s environmental health was an important concern.

**Conclusions:**

We conclude that effective communication of exposure results of unknown clinical significance to participants in environmental health studies can be achieved by providing group summary results and actionable health information. Results suggest that there was an improvement in knowledge of environmental health and in risk reduction behaviors in our study population.

## Background

The goal of many environmental health research studies is to understand the relationship between exposure to environmental toxicants and health outcomes. For studies in humans, environmental exposures may be assessed by recruiting community residents, assessing them for biomarkers of exposure to pollutants and determining any measurable health effects that correlate with this exposure. More recently, environmental health researchers are recognizing that these studies also need to address the health concerns of the study participants and the communities that they represent [1, 2]. One approach to conducting environmental health research studies is termed “community-based participatory research” (CBPR), defined as a modality of research that focuses on collaborating with community partners throughout the entire research process, from developing the research protocol, to conducting analyses, and finally to disseminating research results [2].

### Report back as a health communication component of CBPR

Disseminating research results is an important health communication component of CBPR [3], yet there are several challenges that researchers and IRB members face providing exposure results of environmental health studies to a population of study participants [4]. One such challenge is the uncertainty of the clinical significance of health effects of most environmental exposures. Communicating clinical uncertainty is very difficult in most community-based settings because the usefulness of the environmental exposure information may not be so apparent. A second challenge of communicating exposure results to participants is that receipt of their exposure measures may result in legal obligations under related laws. The extent to which study participants should be provided results of unknown clinical significance is still under debate [5, 6].

#### Arguments against report-back

In applying core principles of research ethics, the decision between autonomy and beneficence comes into play [7, 8]. As described in the National Commission for the Protection of Human Subjects of Biomedical and Behavioral Research’s Belmont Report the principle of autonomy recognizes an individual’s right to self- determination while beneficence is related to actions done for the benefit of others in order to prevent or remove harm [9]. Some Institutional Review Boards (IRBs) have objected to reporting individual biomarker results on the grounds that data with a large degree of clinical uncertainty can affect an individual’s understanding and/or lead to some psychological harm [10]. In these situations, disseminating results may be in violation of the ethical principle of beneficence.

For instance, clinical uncertainty in research results may affect the participant’s ability to understand the results they receive. Shalowitz and Miller describe how if researchers themselves aren’t able to confidently interpret the results, it may be difficult to communicate the uncertainties clearly to participants. Furthermore, scientific information can be complex and difficult for participants to understand in general, which may cause them anxiety or promote them to take unnecessary or harmful steps due to health information that was not understood or was not properly communicated [11].

Another issue related to the uncertainty in the clinical significance of environmental exposure results is whether or not the results have clinical utility. If the results are not clinically useful, this also can result in harm to individuals receiving their biomarker results. A number of prominent U.S. organizations involved in research emphasize that harm can result if the health effects of the environmental exposure is unknown and/or if there are no actions that the participant can take to ameliorate the risk [11]. The view that only clinically significant and useful results be communicated to participants is supported by the National Bioethics Advisory Committee guideline to report biomarkers only when health implications have been established [12].

In reporting back results from environmental exposure studies, there has also been concern about possible legal implications for participants who receive their individual results. For example, exposure results may show high levels of toxic chemicals in a participant’s home that would require disclosure to future buyers or renters of a property [13]. This could potentially violate the ethical principle of beneficence because this requirement would put a burden on the participants that would not have otherwise existed if they did not receive their exposure results.

#### Arguments for report-back

Proponents of reporting research results argue that by not communicating results, researchers deny participants the rights to access information, violates research ethics, and doesn’t take into account that exposure risks are modifiable [10]. The European Union has a Data Protection Directive stating that it is illegal (in most EU countries) to deny participants data obtained through their participation in research [14]. Interpretation of the EU Directive suggests that in the event that results lead to unknown health effects, the researchers should explain the scientific uncertainties to the study participants [15]. Similarly, in CBPR, the participants’ right to access results is based on the belief that the participant owns their personal information regardless of its clinical significance. Additionally, withholding information based on a fear of causing anxiety or worry may unintentionally violate the principles of participants’ autonomy [10]. Moreover, proponents argue that clinical research guidelines cannot be applied to environmental exposure research because withholding information about chemical exposure levels fails to account for some of the exposure risks that are modifiable by individual actions to avoid sources of exposure, unlike other types of clinical research data such as genetic information [10].

### Environmental health communication in vulnerable populations

Trust and health literacy are essential factors to consider when communicating with study participants. When conducting research in minority communities, trust becomes a reoccurring theme that needs to be addressed. Our previous studies have shown that Blacks and Latinos reported a higher level of distrust in research, but they are still just as likely to participate in research as other groups [16]. Furthermore, trust has also been found to be an important factor when vulnerable populations receive health information. The source (i.e. the messenger) of health information must be trusted in order for a population to accept the health information provided to them [17, 18].

While trust is a significant component of health communication, health literacy is another factor to be conscious of when working with vulnerable populations. The National Assessment of Adult Literacy (NAAL) examined the health literacy of U.S. adults classifying literacy levels as below basic, basic, intermediate, and proficient. The assessment found that 36% of adults in the US had health literacy skills at a basic or below basic level [19]. A focus group study on environmental health risks and communication challenges among low socioeconomic status populations and racial/ethnic minorities found that understanding health information was made difficult by an overwhelming quantity of information given, use of complicated language, and presentation of contradictory health information [20].

The purpose of this paper is to present our experience in reporting pollutant exposure information to participants and/or their guardians in a community-based study of environmental exposures and puberty development. In our study, we addressed the ethical challenges to communication in a CBPR study design. Our experience may serve to illuminate the current debate regarding whether and how to report study results to participants in an environmental exposure study.

## Methods

### Study design, Population & Setting

*Growing up Healthy* (GUH) was a longitudinal study that aimed to assess the effects of exposure to endocrine disrupting chemicals, diet, physical activity and characteristics of the built environment on body weight and pace of puberty in a cohort of low-income minority girls [21, 22]. The study was prompted by earlier investigations suggesting that endocrine disrupting chemicals could affect mammary tissue development, potentially predisposing exposed individuals to breast cancer. GUH was one of four Breast Cancer and the Environment Research Centers (BCERCs) conducting coordinated research focused on endocrine disruptors and their effects on girls’ puberty and development [23]. During 2004–2007, GUH at the Icahn School of Medicine at Mount Sinai recruited 416 girls at ages 6–8 years through consent by their parent or legal guardian. All participants lived in East Harlem and other nearby low income New York City neighborhoods. The study population was 75% Latino and 38% African-American. Spanish was the household language in 43% of the study’s parents. In the overall cohort, 54.5% of families reported household incomes under $25,000 and 33.6% of the parents had less than a high school diploma. Thirty percent of the girls were from immigrant families newly arrived from the Puebla region of Mexico. Participants recruited were followed for seven years after recruitment to assess the relationship between environmental exposures and pace of puberty.

### Intervention

Girls enrolled in the study were evaluated for pubertal development at baseline and then annually. Development was assessed through breast staging and examination of pubic hair by a pediatrician or nurse practitioner. Urine and blood samples were collected for measurement of biomarkers of exposure to endocrine disrupting chemicals. There were four classes of endocrine disrupting chemicals that were assessed were: (phthalates, phenols, parabens, and phytoestrogens). Specifically, samples were assessed for exposure to Phenols (benzophenone-3, enterolactone, bisphenol A, methyl-, ethyl-, propyl-parabens, 2,5-dichlorophenol, triclosan, genistein, and daidzein). Phthalate metabolites (monoethyl phthalate (MEP), mono-*n*-butyl phthalate (MBP), mono-isobutyl phthalate, monobenzyl phthalate (MBzP), mono-3-carboxypropyl phthalate (MCPP), mono(2-ethyl-5-carboxypentyl) phthalate, mono(2-ethyl-5-hydroxylhexyl) phthalate (MEHHP), mono(2-ethyl-5-oxohexyl) phthalate (MEOHP), and mono(2-ethylhexyl) phthalate (MEHP)). Phytoestrogens (flavonol and lignin). And paraben metabolites, which were grouped based on molecular weight (micromoles/L), expressing the paraben sum as propyl paraben (molecular weight 180.2 g/mol). Parents completed annual questionnaires on dietary habits, use of personal care products and physical activity, which was administered by telephone [24, 25].

Exposure levels to these chemicals were then compared to exposure levels of a national sample of children of the same ages collected in the annual National Health and Nutrition Examination Survey (NHANES). For the purpose of the present study, we focused on reporting back to the participants the results for Mono-2-ethylhexyl phthalate as a compound representative of exposure to phthalates and because comparable data existed from the NHANES studies [26]. At baseline, the cohort was found to have levels of this phthalate that were higher than those reported by NHANES for children of the same age (5.3 ng/ml compared to national average of 4.4 ng/ml).

### Community collaboration

The Community Outreach and Translation Core (COTC) was responsible for recruitment and retention of study participants and creating strategies for communicating with parents [26]. The COTC created a fifteen-member Community Advisory Board (CAB) composed of representatives from the local school district, community youth organizations, social service agencies, community health centers, the local public hospital, WIC programs, an environmental justice organization, the local office of the New York City Department of Health, community residents/leaders from the local community planning board and parents of study participants. The CAB provided guidance to investigators on study protocol throughout recruitment, enrollment and retention. They advised the researchers on communication methods to reach both participants and the larger community that were culturally appropriate, understandable to lay audiences with low literacy in English, and tailored to neighborhood-specific concerns.

### Rationale for communication of exposure study results

Together, the investigators and the CAB decided to provide summary results of biomarker exposures to the parents of study participants instead of individual-level results. This was in alignment with the position of the Mount Sinai School of Medicine Institutional Review Board primarily based on the uncertainty of what the exposure results would mean on an individual basis and the absence of evidence linking exposures to specific health outcomes including the specific health outcomes being studied.

Exposure results for the entire cohort were presented to the CAB by the study team with comparisons to the NHANES data [27]. This initial report was criticized as “too technical” for most community residents and study participants. The CAB members were concerned that presenting data with this technical approach could alarm or scare participants. They strongly supported an educational approach to communicating exposure levels and recommended that the format be changed to use more graphic visuals. Members of the CAB also recommended the inclusion of messages of how to reduce risk and how to use available alternatives to products that might be sources of exposure to phthalates. Questions regarding the “right to know” or the “right not to know” the results of chemical exposure levels were not raised by the CAB members. They were more concerned about educating families in the absence of known cause and effect.

In response to this feedback, a newsletter format was developed collaboratively by the researchers in the COTC and CAB members. The newsletter provided group-level exposure results and more information about chemical/phthalate exposure and how to avoid it. The COTC and CAB designed the newsletter to include: 1) the use of visuals 2) was targeted to a 4th grade literacy level, 3) focused on prevention and alternatives to products and 4) included no more than two figures of data, 5) included information of researchers that participants could contact for additional information or to access their child’s exposure levels.

**Fig. 1:**
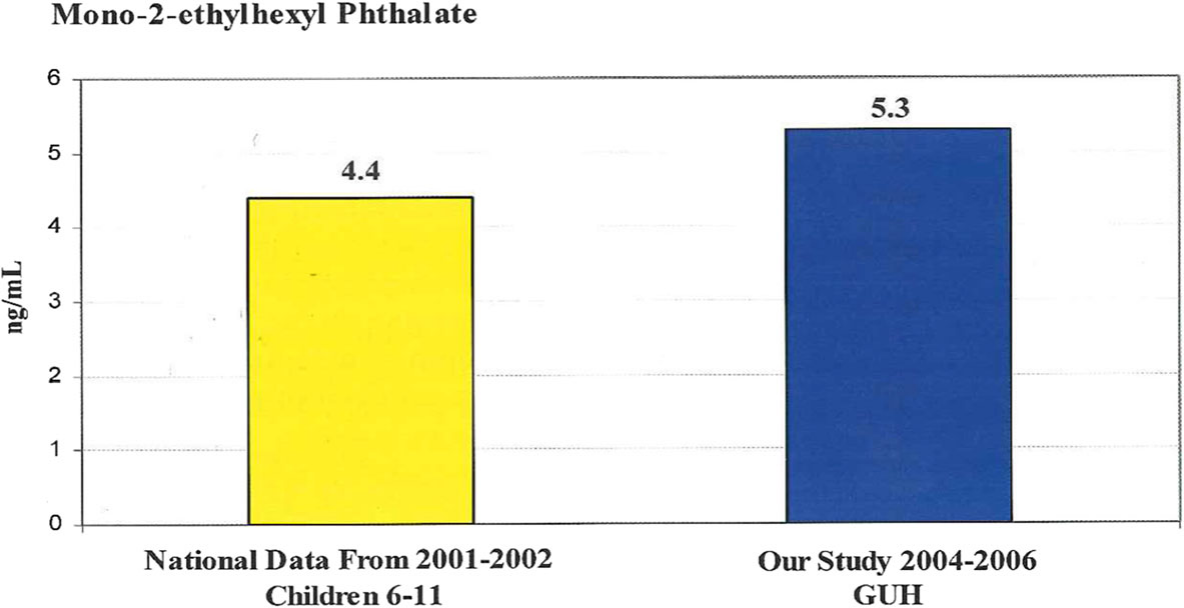
Graph and information as presented to study participants. Title, graph and figure legend as provided to study participants which showed average urinary levels of mon-2-ethylhexyl phthalate in study participants as compared with a national cohort from the NHANES Second National Report on Human Exposure to Environmental Chemicals

Based on the CAB’s suggestions to focus on prevention and actions that could potentially reduce exposure, three iterations of the info-graphic were tested with focus groups of three or more study participants and their input was incorporated into the final newsletter to be distributed to all study participants. The final newsletter was written in Spanish and English and included information on phthalates, suggestions of actions that may reduce exposure, group summary results (Fig. 1), and an invitation to ask for more information with staff telephone numbers.

### Assessing effectiveness of the newsletter

After distribution of the newsletter and the group biomarker results, a 14-item questionnaire was mailed to 310 study participants who had results on exposure to mono-2-ethylhexyl phthalate. The questionnaire was designed to assess participants’ comprehension and knowledge of the health information presented in the newsletter, in particular the data on exposure to mono-2-ethylhexyl phthalate (Fig. 1). At the request of the CAB, we removed the standard deviation or standard error bars in order to present a clearer message to the participants. We included four “getting to know more about you” questions to understand the preferences and interests in environmental health of the respondents. The questions were a combination of multiple choice and yes/no questions with an “I don’t know option”. The questions are listed in Table 1.

**Table 1:** Questionnaire

1) What types of plastics are believed to be safe for people?	
2) Why should you avoid some plastics?	
3) How can you avoid phthalates?	
4) Do you feel you need more information about phthalates?	
5) Will your shopping list or product use change after reading the newsletter about phthalates in plastics?	
6) Will you be looking for the recycle number on your next shopping trip?	
7) What was the most helpful information in the newsletter?	
8) What did you understand about the graph on page #3 of the newsletter?	
9) Did you hear or learn about phthalates in plastics before reading the newsletter?	
10) If you have heard or learned about phthalates in plastics before, where did you learn this information?	
^a^11) What are your most important concerns about your children’s health?	
^a^12) What topics do you want to learn more about?	
^a^13) My relation to the child participating in the Growing Up Healthy study is…	
^a^14) How do you prefer to receive information from this program?	

Note. ^a^ Getting to know you questions

## Results

Questionnaires were sent to 310 households and we received 106 responses; a 34% response rate. Most responders to the questionnaire were mothers of the girls participating in GUH (93%). The demographic characteristics of the responders were comparable to the general study participant population: 51% were Spanish speaking and 49% were English speaking while the general study population was 43 and 57%, respectively.

### Improvement in environmental health literacy among study participants

Our results show improvement in our survey population’s knowledge about environmental health. For example, in response to the comprehension question “What types of plastics are believed to be safe for people?” 63% of Spanish responders and 73% of English speakers responded correctly (Table 2). Moreover, 83% of the Spanish speakers and 98% of the English speakers recognized the main message that some plastics should not be used to store food. Most responders also identified multiple ways in which exposure to phthalates can be reduced with comparable response between the English and Spanish speaking groups.

**Table 2:** Percentage of Responders

	All responders *n* = 106		Spanish-Speakers *n* = 54		English-Speakers *n* = 52	
	Correct	Incorrect or Don’t know	Correct	Incorrect or Don’t know	Correct	Incorrect or Don’t know
What types of plastics are believed to be safe for people?	69%	31%	65%	35%	73%	27%

Overall, 73% of responders said that they had not heard of or learned about phthalates in plastics before reading the newsletter. Those who had heard about phthalates noted various sources of information, including other GUH events and communications, newspapers, television, and the internet.

Ninety eight percent of the population preferred receiving results through the mail. Responders stated that the sections of the newsletter that were most informative were: steps to lower contact with phthalates (41%), followed by information about safety of phthalates (34%), and the study findings on levels of exposure in the participants (21%).

### Risk reduction strategies among study participants

We found that the information provided in the Newsletter was used by parents to address potential phthalate exposure. We found that 83% of participants (88% Spanish speaking and 77% English speaking) chose to change some product use habit after reading the newsletter. For example, 91% of the total participants (96% Spanish speaking and 85% English speaking responders), reported that they use the recycling number on plastic containers to identify the plastic types and reduce exposures to potentially toxic components.

### Interest in environmental health among study participants

Respondents expressed environmental health as an important concern for their child’s health and a desire for more environmental health information. Table 3 shows which topics responders consider to be most important about their children’s health, these included environmental health (36%), puberty at a young age (31%), and obesity (28%). Spanish-speaking and English-speaking responders noted environmental health and puberty in equal proportions. Interestingly, Spanish-speaking responders (67%) represented a larger percentage of people who said that obesity was their primary concern, compared to the English-speaking responders (38%). Participants also stated that they wanted to learn more about child growth and development (35%), alternatives to toxic products (30%), and environmental health (29%). Additionally, results from the questionnaire revealed that 89% Spanish-speaking and 71% English-speaking responders would like more information specifically on phthalates.

**Table 3:** Percentage of Responders

What are your most important concerns about your child’s health?	All responders *n* = 106	Spanish-Speakers *n* = 54	English-Speakers *n* = 52
Obesity	52.8	66.6	38.4
Puberty at a young age	59.4	61.1	57.6
Environmental Health	67.9	64.8	71.1
No concerns	3.7	7.4	0
Other	3.7	1.8	5.7
Don’t know	1.8	1.8	1.9

## Discussion

Disseminating results to participants of environmental exposure studies is not widely practiced due to concerns of clinical significance of such results at the individual level. However, studies have shown that communicating individual results can be beneficial to participants; such as increasing environmental health awareness and motivating participants to reduce environmental exposures [27–30]. Our study addressed the potential ethical challenges to communication by incorporating community collaboration, providing group summary results, and providing actions for reducing environmental exposure.

### Collaborating with the community advisory board to establish an effective communication strategy with research participants

We consulted with the CAB to ensure that our communication strategy would be appropriate and effective for our study population. The study was implemented with the belief that it is our responsibility as researchers to communicate results to research participants in a way that is understandable to them despite the clinical uncertainty in the exposure results. Feedback from the newsletter questionnaire and guidance from the community leaders (CAB) was critically important to study investigators’ understanding of ongoing communication with the parents of the study population. Moreover, a research study that must rely on community participation over a long term can only benefit from sharing decision making with community leaders and research participants. With the CAB’s support and guidance, we used an educational approach to understanding exposure levels and ultimately were able to communicate the information in an appropriate manner for the study participants and the community at large; allowing for successful report back of exposure results. Our partner group at the University of Cincinnati (Growing Up Female) also studied the issue of providing exposure results to participants during their pilot phase, and focused on how to develop a communication plan for the study participants. The authors formed an advisory committee of stakeholders who worked through the issues between the “right to know” and “beneficence” ultimately choosing to report the results as well [31].

### Providing group exposure results compared to national averages instead of individual exposure results to address concerns regarding the unknown clinical significance

We provided group exposure results compared to national averages instead of individual exposure results. In presenting group data, the study was able to potentially reduce the harm of creating stress for the community, or for an individual family regarding the phthalate exposure levels in their child. Furthermore, communicating the group exposure results as compared to national averages helped to give context to the results. Guidance based on previous environmental health communication studies have also suggested providing group exposure results with comparisons to similar groups to allow participants to better understand their exposure results in the context of other studies [32].

### Providing empowering actions and information for participants to take to reduce their exposure to chemicals

In communicating results with unknown clinical significance, we addressed the possibility of causing undue concern among participants regarding what they may be able to do about their phthalate levels by providing empowering actions that they could take to potentially reduce exposure. A recent study showed a small reduction in exposure biomarkers to phthalates in adolescent girls when given products that contained lesser amounts of these chemicals [33]. Our questionnaire results suggested that parents were interested in taking steps to avoid phthalate exposure. Such response has been shown in other exposure studies that have utilized individual results communication in minority and environmental justice communities and reported comprehension of complex scientific material along with behavior changes to reduce exposure [28, 29]. Our study demonstrates that communicating group study results is also effective in facilitating behavioral changes to reduce environmental exposures. In addition to facilitating behavioral changes, CBPR utilizing report back methods may serve as a way to improve environmental health literacy. Ramirez-Andreotta et al. describe how informal science education can increase environmental health knowledge in communities affected by environmental issues [34].

### Limitations

The results of this study are limited by sample size. We found that responders were not different in terms of demographics to the general study population and could not find any ways in which responders may have been different than non-responders. However, it is possible that the people who responded were more interested in learning about the study and environmental health than those that did not respond. we were able to build a relationship of trust with our study participants over a period of several years. It is also important to note that our study was longitudinal in design and our communication and report back strategy may not be appropriate for other study designs.

## Conclusions

Over the last few years the practice of report-back has slowly increased and major guidance documents from organizations including the Environmental Protection Agency and the National Academies of Sciences have called for report-back [32]. Although crucial to community-based participatory research, report back is not always practiced in environmental health exposure assessment studies. We recommend that it should be strongly considered. We found that we were able to increase environmental health knowledge and provide information for participants to reduce their environmental exposure risk. Our results suggests that communicating environmental health information and risk reduction strategies along with group summary results can address the concerns surrounding report back of environmental exposure levels to study participants.
